# Complications and Risks Associated With the Different Types of Abdominoplasties: An Analysis of 55,956 Patients

**DOI:** 10.1093/asj/sjae060

**Published:** 2024-03-18

**Authors:** Sara C Chaker, Ya-Ching Hung, Mariam Saad, Galen Perdikis, James C Grotting, K Kye Higdon

## Abstract

**Background:**

Different types of abdominoplasties have been developed to address individual patient characteristics. However, an analysis of complication rates and risk factors for different types of abdominoplasties has yet to be reported.

**Objectives:**

The aim of this study was to evaluate the complication rates and risks associated with each type of abdominoplasty.

**Methods:**

Utilizing the CosmetAssure database, patients undergoing an abdominoplasty from 2015 to 2022 were identified. Demographic factors and major complications were recorded and analyzed with a chi-square test or analysis of variance. A logistic regression was performed to identify the risk for developing complications associated with each type of abdominoplasty.

**Results:**

A total of 55,596 patients underwent an abdominoplasty procedure by any method. The overall complication rate was 2.1%. There was a significant difference in the overall complication rates of all 7 types of abdominoplasties (*P* < .05), with fleur-de-lis abdominoplasty having the highest complication rate. The year of surgery, being underweight or morbidly obese, having diabetes, and being male placed patients at a significantly higher risk for developing a postoperative complication. Over 15,000 patients (27.2%) had concurrent procedures related to breast surgery, other body contouring, liposuction, or facial surgery. When accounting for various risk factors in a regression model, there was no significant added risk for major complications after a combination procedure with an abdominoplasty compared to abdominoplasty alone.

**Conclusions:**

Among the different types of abdominoplasties, a fleur-de-lis abdominoplasty has the highest complication rate. Concurrent cosmetic procedures with an abdominoplasty showed no added risk for major complications when compared to abdominoplasty alone.

**Level of Evidence: 3:**

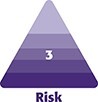

According to The Aesthetic Society (formerly ASAPS), abdominoplasty, or “tummy tuck,” was the third most common cosmetic surgical procedure performed in 2022 by plastic surgeons, behind liposuction and breast augmentation, with 207,262 procedures completed.^1^ In fact, abdominoplasty has consistently ranked in the top 5 cosmetic procedures performed in the US over the last decade.^1,2^ Patients often elect to undergo an abdominoplasty following substantial weight loss or pregnancy, or to remove excess abdominal skin due to loss of elasticity or aging.^3^ The popularity of this elective cosmetic procedure stems from its ability to provide positive psychological effects for patients who undergo the procedure.^4,5^ It has been reported that among the most popular cosmetic plastic surgery procedures abdominoplasty has the highest patient-reported satisfaction.^6^

Since the development of the procedure in the 1970s, numerous different types of abdominoplasties have been developed to address different patient needs and characteristics.^3^ The conventional or traditional abdominoplasty remains the most common procedure for patients who have excess or sagging skin above and below the umbilicus.^7^ A lipoabdominoplasty includes extensive abdominal liposuction concurrent with a conventional abdominoplasty procedure.^7,8^ A mini abdominoplasty is a shorter incision variation and is usually recommended for patients with a smaller area of skin laxity only in the infraumbilical abdomen.^9^ A circumferential abdominoplasty is a more extensive procedure addressing the area above and below the umbilicus anteriorly, but also carrying circumferentially around the lower back or flanks to include the area above the intergluteal cleft.^10^ An extended abdominoplasty extends the lateral margins of excision of the conventional abdominoplasty to the lateral hips and flanks.^11^ A reverse abdominoplasty is a technique typically targeting excess or loose skin in the upper portion of the abdomen and requires an inframammary incision placement.^12^ Last, a fleur-de-lis abdominoplasty is a more complex procedure often for postbariatric patients or other forms of massive weight loss and involves a vertical incision along the midline of the abdomen in addition to that performed in abdominoplasties of the conventional, extended, and other types.^13^

Despite the many surgical advancements and variations in an abdominoplasty technique, postoperative complications after abdominoplasty have been shown to be the highest compared to all other cosmetic procedures.^14,15^ Due to factors inherent in a patient population seeking body contouring and the degree of invasiveness of this procedure, the complication rate of a traditional abdominoplasty in some studies has been reported at as high as 51.8%.^16-18^ Yet other authors have shown that abdominoplasty can have a low complication rate, as low as 3.1% when performed as a solo procedure and 4% when combined with other procedures.^15^ Despite the evidence for safety of abdominoplasty in general, the individual complication rates of the different types of abdominoplasties have yet to be reported in the literature. Because each type of abdominoplasty is recommended based on individual patient characteristics and their desired aesthetic outcomes, it is important to provide information on the associated risks with each type of abdominoplasty procedure.

In this study we aimed to delineate the complication rates and risk factors specific to the various types of abdominoplasties within a large national database that was validated for cosmetic surgery procedures.

## METHODS

This study was approved by the Vanderbilt University Institutional Review Board (IRB# 230893). Informed consent was not required for this study because data obtained from the CosmetAssure (Birmingham, AL) database is deidentified.

### Patient Population

The study population consisted of patients who were prospectively enrolled into the CosmetAssure database and underwent a cosmetic surgical procedure(s) between March 2015 and December 2022. Patients who underwent any type of abdominoplasty from between March 2015 and December 2022 and were covered by CosmetAssure insurance were included in the analysis. Patients with more than 1 type of abdominoplasty were grouped to the most invasive type of procedure. For example, if a patient underwent a fleur-de-lis and an extended abdominoplasty, the patient was categorized as having a fleur-de-lis abdominoplasty. The order of categorization was as follows: fleur-de-lis > circumferential abdominoplasty > extended abdominoplasty > conventional abdominoplasty > reverse abdominoplasty > lipoabdominoplasty > mini abdominoplasty. Query of the database was initiated in May 2023 and conducted by S.C.C. Both S.C.C. and Y.C.H. reviewed the results, and any disagreements were resolved through discussion between the 2 authors or discussion with the senior author (K.K.H.).

### Database

CosmetAssure is an insurance program that offers financial coverage for unanticipated costs associated with complications when they occur in patients undergoing elective cosmetic surgical procedures. First introduced in 2003, CosmetAssure covers major complications accrued for 27 common cosmetic procedures, which may or may not be covered by a patient's primary health insurance. The program is offered in all 50 states of the United States and is endorsed by the American Society of Plastic Surgeons (ASPS) and The Aesthetic Society. It is available exclusively to American Board of Plastic Surgery (ABPS)–certified plastic surgeons and to membership candidates for the ASPS and The Aesthetic Society who have passed the ABPS written examination. The CosmetAssure program requires all surgical procedures completed by approved surgeons to be performed in accredited facilities. Patients who undergo 1 or more of the covered procedures by a participating plastic surgeon at an accredited facility are entered into the database before any procedure, making this database a prospective patient repository. Surgeon-reported major complications, filed as a claim, are recorded in the database. A major complication is defined as one occurring within 45 days of the operation and requiring a hospital admission, an emergency room visit, or a reoperation. This excludes minor complications that can be managed in the clinic such as minor wound infection or minor seroma, because they are not eligible for an insurance claim. The major complications covered by CosmetAssure include arrhythmia, cardiac arrest, deep vein thrombosis (DVT), myocardial infarction, pulmonary embolus (PE), capsular contracture, and anesthesia-related complications such as severe hypotension and hypertension. Surgical-related complications include capsular contracture, hematoma, hemorrhage, significant skin necrosis, and infection. The database also records other variables, including all concurrent surgical procedures a patient undergoes. Demographic and comorbidity data recorded includes age, gender, body mass index (BMI), smoking status, diabetic syatus, year of surgery, type of anesthesia given, American Society of Anesthesiologists (ASA) classification system physical status, and type of surgical facility (office-based surgery center, ambulatory surgery center, hospital).

### Outcomes

**Figure 1.**
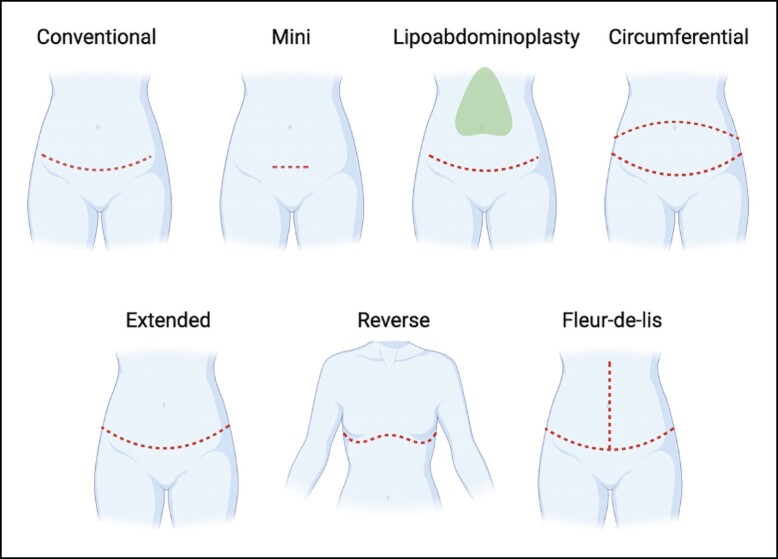
Incision markings for each type of abdominoplasty. Illustration created with Biorender.com (Toronto, Ontario).

The primary outcome included any complications such as PE, DVT, hematoma, infection, hypoxia, pulmonary dysfunction, hypotension, fluid overload, arrhythmia, myocardial infarction, shock, cardiac arrest, or hypertension. We included 7 types of abdominoplasty in our analysis, as follows: conventional abdominoplasty, lipoabdominoplasty, mini abdominoplasty, circumferential abdominoplasty, extended abdominoplasty, reverse abdominoplasty, and fleur-de-lis/inverted-T excision (Figure 1). Secondarily, complication rates of an abdominoplasty in combination with other cosmetic surgery procedures were investigated. Cosmetic surgery procedures were grouped as follows: breast procedures (augmentation, mastopexy, reduction, implant revisional procedures, treatment of gynecomastia); liposuction; body contouring procedures (gluteal augmentation, thigh lift, brachioplasty, vaginoplasty); and facial procedures (blepharoplasty, brow lift, facelift, rhinoplasty, septoplasty, otoplasty). Any cosmetic procedure determined not appropriate for placement in any of these categories was labeled as “other.”

### Statistical Analysis

Descriptive statistics were performed with a chi-square test for categorical variables and analysis of variance for continuous variables. For purpose of univariate analysis, BMI was recoded as a categorical variable with clinically appropriate categories (underweight <18, normal 18-25, overweight 25-30, obese 30-35, severe obesity 35-40, and morbid obesity >40). Independent risk factors for postoperative complications for each type of abdominoplasty were identified with logistic regression. A *P* value less than .05 2-tailed determined statistical significance. All statistical analyses were conducted with Stata SE statistical software, version 18.0 (StataCorp LP, College Station, TX).

## RESULTS

From March 2015 to December 2022, a total of 251,402 patients were captured in the CosmetAssure database. Of these patients, 55,596 patients underwent an abdominoplasty procedure. The average age at procedure was 43 years old (interquartile range = 36-51) and nearly all the patients were female (female, *n* = 53,803 [96.8%]; male, *n* = 1793 [3.2%]). There was an overall complication rate of 2.1%. Nearly 40% of the surgeries occurred in 2021 and 2022. All demographic information is presented in Table 1.

**Table 1.** Patient Demographics

*Variable*	*Abdominoplasty (n = 55,596)*
Age, years; median (IQR)	43 (36, 51)
Smoker	
No	53,556 (96.3%)
Yes	2040 (3.7%)
Diabetic	
No	54,085 (97.3%)
Yes	1511 (2.7%)
Gender	
Female	53,803 (96.8%)
Male	1793 (3.2%)
BMI, kg/m^2^	
Underweight (<18)	56 (0.10%)
Healthy (18-25)	9070 (16.3%)
Overweight (25-30)	18,100 (32.6%)
Obese (30-35)	15,643 (28.1%)
Severely obese (35-40)	7963 (14.3%)
Morbidly obese (>40)	4764 (8.6%)
Year of surgery	
2015	4123 (7.4%)
2016	5521 (9.9%)
2017	5658 (10.2%)
2018	6118 (11.0%)
2019	6650 (12.0%)
2020	6852 (12.3%)
2021	10,598 (19.1%)
2022	10,076 (18.1%)
Overall complications	
None	54,416 (97.9%)
Yes	1180 (2.1%)

BMI, body mass index; IQR, interquartile index.

### Type of Abdominoplasty

Of the 55,596 patients who underwent an abdominoplasty, 40,493 underwent a conventional abdominoplasty, 5300 patients had an extended abdominoplasty, 4190 underwent a lipoabdominoplasty, 3983 patients underwent a mini abdominoplasty, 761 patients underwent a fleur-de-lis/inverted-T excision procedure, 721 had a circumferential abdominoplasty, and 148 patients underwent a reverse abdominoplasty (Table 2). The majority of patients who underwent a conventional abdominoplasty (33.1%), lipoabdominoplasty (32.3%), mini abdominoplasty (36.7%), and reverse abdominoplasty (31.1%) were overweight (BMI 25-30), and the majority of patients who underwent an extended abdominoplasty (28.7%), a fleur-de-lis/inverted-T excision procedure (28.5%), or a circumferential abdominoplasty (30.4%) were obese (BMI 30-35) (Table 2).

**Table 2.** Complication Rates by BMI and Type of Abdominoplasty

BMI, kg/m^2^	*Conventional abdominoplasty (n = 40,493)*	*Extended abdominoplasty* *(n = 5300)*	*Lipoabdominoplasty* *(n = 4190)*	*Mini abdominoplasty* *(n = 3983)*	*Fleur-de-lis/*i*nverted*-*T excision* *(n = 761)*	*Circumferential abdominoplasty* *(n = 721)*	*Reverse abdominoplasty* *(n = 148)*	*P value*
Underweight (<18)	40 (0.10)	4 (0.08)	0	10 (0.25)	0	1 (0.14)	1 (0.68)	.004
Healthy (18-25)	6499 (16.1)	531 (10.0)	670 (16.0)	1165 (29.3)	63 (8.28)	84 (11.7)	58 (39.2)	<.001
Overweight (25-30)	13,401 (33.1)	1432 (27.0)	1352 (32.3)	1460 (36.7)	195 (25.6)	214 (29.7)	46 (31.1)	<.001
Obese (30-35)	11,636 (28.7)	1519 (28.7)	1186 (28.3)	842 (21.1)	217 (28.5)	219 (30.4)	24 (16.2)	<.001
Severely obese (35-40)	5733 (14.2)	1004 (18.9)	596 (14.2)	320 (8.03)	162 (21.3)	184 (18.6)	14 (9.46)	<.001
Morbidly obese (>40)	3184 (7.86)	810 (15.3)	386 (9.21)	186 (4.47)	124 (16.3)	69 (9.57)	5 (3.38)	<.001

Values provided are complication rates, with percentage in parentheses. BMI, body mass index.

**Figure 2.**
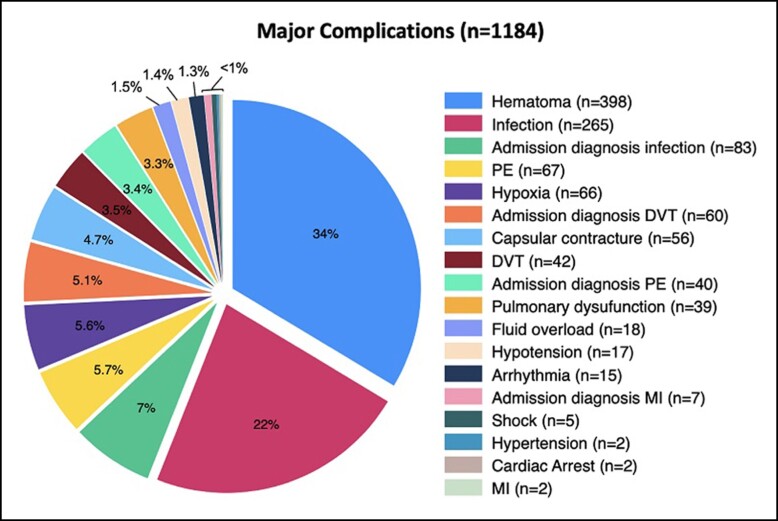
Pie chart of all major complications. DVT, deep vein thrombosis; MI, myocardial infarction; PE, pulmonary embolism.

There was a significant difference between overall complication rates of the different types of abdominoplasty procedures (*P* = .015). The procedure that had the lowest overall complication rate was a reverse abdominoplasty (1.35%), while patients undergoing a fleur-de-lis abdominoplasty presented the highest overall complication rate of 3.81% (Table 3). Patients older than 60 years showed an increased rate of complications in nearly all types of abdominoplasties, except in the case of reverse abdominoplasty (Table 4). Hematoma and infection were the greatest contributors to the overall number of complications across all types of abdominoplasties (34% and 22%, respectively; Table 3, Figure 2).

**Table 3.** Incidence of Specific Complications by Type of Abdominoplasty

	*Conventional abdominoplasty (n = 40,493)*	*Extended abdominoplasty* *(n = 5300)*	*Lipoabdominoplasty* *(n = 4190)*	*Mini abdominoplasty* *(n = 3983)*	*Fleur-de-lis/*i*nverted T*-*excision* *(n = 761)*	*Circumferential abdominoplasty* *(n = 721)*	*Reverse abdominoplasty* *(n = 148)*	*P value*
Overall complications	855 (2.11)	131 (2.47)	78 (1.86)	72 (1.81)	29 (3.81)	17 (2.36)	2 (1.35)	.008
Hematoma	282 (0.70)	43 (0.81)	26 (0.62)	29 (0.73)	9 (1.18)	8 (1.11)	1 (0.68)	.50
Infection	199 (0.49)	28 (0.53)	12 (0.29)	13 (0.33)	10 (1.31)	3 (0.42)	0	.007
Admission diagnosis infection	55 (0.14)	7 (0.13)	5 (0.12)	9 (0.23)	4 (0.53)	3 (0.42)	0	.038
Confirmed PE	49 (0.12)	10 (0.19)	5 (0.12)	1 (0.03)	2 (0.26)	0	0	.286
Admission diagnosis PE	32 (0.08)	4 (0.08)	3 (0.07)	0	1 (0.13)	0	0	.655
Hypoxia	47 (0.12)	7 (0.13)	4 (0.10)	5 (0.13)	1 (0.13)	2 (0.28)	0	.917
DVT	29 (0.07)	5 (0.09)	3 (0.07)	4 (0.10)	1 (0.13)	0	0	.950
Admission diagnosis DVT	47 (0.12)	9 (0.17)	3 (0.07)	1 (0.03)	0	0	0	.326
Pulmonary dysfunction	28 (0.07)	6 (0.11)	4 (0.10)	1 (0.03)	0	0	0	.665
Hypotension	12 (0.03)	2 (0.04)	2 (0.05)	0	0	1 (0.14)	0	.575
Fluid overload	10 (0.02)	3 (0.06)	1 (0.02)	3 (0.08)	0	0	1 (0.68)	.001
Arrhythmia	10 (0.02)	3 (0.06)	1 (0.02)	1 (0.03)	0	0	0	.894
MI	1 (<0.01)	0	1 (0.02)	0	0	0	0	.503
Admission diagnosis MI	5 (0.01)	1 (0.02)	1 (0.02)	0	0	0	0	.972
Shock	4 (0.01)	0	1 (0.02)	0	0	0	0	.915
Hypertension	1 (<0.01)	0	1 (0.02)	0	0	0	0	.503
Cardiac arrest	2 (<0.01)	0	0	0	0	0	0	.93

Percentages are provided in parentheses. DVT, deep vein thrombosis; MI, myocardial infarction; PE, pulmonary embolism.

**Table 4.** Complication Rate by Demographics and Type of Abdominoplasty

	*Conventional abdominoplasty (n = 40,493)*	*Lipoabdominoplasty* *(n = 4190)*	*Mini abdominoplasty* *(n = 3983)*	*Circumferential abdominoplasty* *(n = 721)*	*Extended abdominoplasty* *(n = 5300)*	*Reverse abdominoplasty* *(n = 148)*	*Fleur-de-lis/*i*nverted*-*T excision* *(n = 761)*	*P value*
Age, years								
<40	1.42	1.28	2.03	0.54	2.09	6.67	0.79	.102
41-59	1.73	1.26	1.17	2.37	1.90	0	2.28	.302
*≥*60	3.26	2.79	2.45	3.57	3.43	1.19	6.25	.024
Diabetes								
Yes	3.62	3.70	1.49	4.35	2.05	0	1.59	.817
Gender								
Male	3.52	1.22	3.75	1.48	3.70	0	5.41	.739
Female	2.07	1.85	1.68	2.56	2.41	1.39	3.73	.009
Smoking status								
Yes	2.69	2.82	1.48	3.03	2.54	0	5.71	.891
BMI, kg/m^2^								
Underweight (<18)	5.0	NA	0	0	0	0	NA	.934
Healthy (18-25)	2.34	1.64	2.32	3.57	2.26	0	4.76	.541
Overweight (25-30)	1.95	1.70	1.78	2.80	2.03	0	3.59	.524
Obese (30-35)	2.01	2.36	1.07	2.28	2.30	4.17	4.61	.042
Severely obese (35-40)	2.09	1.85	1.88	0.75	2.79	7.14	4.32	.187
Morbidly obese (>40)	2.61	1.04	2.15	2.90	3.33	0	1.61	.391
Type of facility								
Office-based surgical center	2.01	2.07	1.84	1.22	2.51	0	1.39	.304
Accredited surgical center	2.15	2.86	2.06	6.35	2.87	0	6.25	<.001
Hospital	2.15	1.69	1.78	2.43	2.41	2.08	5.24	.640
Type of anesthesia								
General anesthesia	2.60	2.33	2.23	2.99	2.80	1.35	4.14	.235
IV sedation	2.87	0	2.52	0	3.03	10.0	0	.844
Local anesthesia	0	0	0	0	0	NA	NA	—
Conscious sedation	0	NA	0	NA	NA	NA	NA	—
ASA classification								
ASA I	3.07	2.05	2.75	0	4.31	3.45	4.71	.132
ASA II	2.66	1.39	1.77	2.44	1.92	0	5.56	.920
ASA III	2.68	12.5	4.35	0	0	0	0	.772
ASA IV	3.49	0	0	0	0	0	0	.976
ASA V	0	0	0	NA	0	NA	NA	—
ASA VI	0	NA	0	NA	NA	NA	NA	—

Values provided are complication rates. ASA, American Society of Anesthesiologists; BMI, body mass index; IV, intravenous; NA, not available.

Our logistic regression model showed that males had a 2 to 3 times higher chance of developing a complication compared to females following a conventional abdominoplasty, mini abdominoplasty, or an extended abdominoplasty (odds ratio [OR], 2.2, 3.1, 2.3, respectively; *P* < .01). Furthermore, patients with a BMI greater than 40 or who were diabetic had a significantly higher risk of developing a complication following a conventional abdominoplasty (OR 1.70, 1.80, respectively) (Table 5). Undergoing a conventional abdominoplasty or a lipoabdominoplasty in 2017, 2018, or 2019 placed patients at greater risk of developing a complication, compared to patients who had surgery performed in 2015. Undergoing a mini abdominoplasty in 2017 or 2019 or an extended abdominoplasty in 2017 or 2018 placed patients at a significantly greater risk of developing a postoperative complication in this cohort (Table 5).

**Table 5.** Regression Model for Overall Complications by Type of Abdominoplasty

Variable	Conventional abdominoplasty (*n* = 40,493)	Lipoabdominoplasty (*n* = 4190)	Mini abdominoplasty (*n* = 3983)	Circumferential abdominoplasty (*n* = 721)	Extended abdominoplasty (*n* = 5300)	Fleur-de-lis/inverted-T excision (*n* = 761)
Age	1.00	0.98	0.99	1.03	0.99	1.01
Smoker (ref nonsmoker)	1.11	2.45	1.27	2.64	1.11	11.6
Diabetic (ref nondiabetic)	1.80	0.71	NA	5.00	0.69	NA
Gender (ref male)	0.46	NA	0.32	NA	0.43	NA
Year of surgery (ref 2015)						
2016	NA	NA	NA	NA	NA	NA
2017	3.61	21.3	10.6	5.59	6.26	NA
2018	4.02	15.3	4.10	1.63	3.80	NA
2019	1.92	6.07	3.62	NA	0.84	NA
2020	0.62	1.47	2.11	NA	0.47	0.15
2021	NA	NA	NA	NA	NA	0.32
2022	NA	NA	NA	NA	NA	NA
BMI, kg/m^2^ (ref healthy)						
Underweight (<18)	21.50	NA	NA	NA	NA	NA
Overweight (25-30)	1.11	0.66	0.99	2.11	0.86	NA
Obese (30-35)	1.34	0.85	0.43	2.69	0.95	2.48
Severely obese (35-40)	1.44	0.50	0.89	NA	1.63	1.25
Morbidly obese (>40)	1.70	0.18	0.99	5.15	1.10	NA
Abdominoplasty plus another procedure	1.14	2.57	1.69	1.74	1.51	2.10
Type of facility (ref hospital)						
Office-based surgery center	0.93	2.00	0.66	5.79	0.80	NA
Accredited surgical center	1.04	0.64	1.22	4.66	1.13	NA
Type of anesthesia (ref general anesthesia)						
IV sedation	1.11	NA	1.06	NA	1.57	NA
Local anesthesia	NA	NA	NA	NA	NA	NA
Conscious sedation	NA	NA	NA	NA	NA	NA

Values provided are odds ratios. BMI, body mass index; IV, intravenous; NA, not available.

### Overall Major Complications

**Figure 3.**
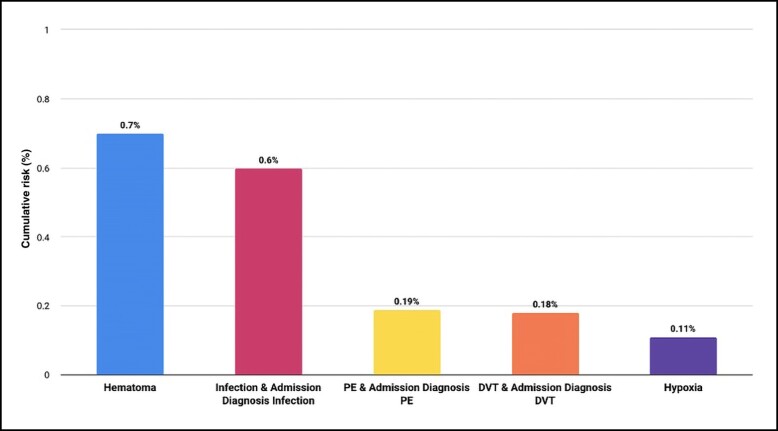
Cumulative risk of the top 5 complications. DVT, deep vein thrombosis; PE, pulmonary embolism.

The overall top 5 major complications among all types of abdominoplasties were similar to those reported in our previous work and are included in Figure 3.^15^ These major complications included: hematoma (0.7%); infection (0.6%), which comprised confirmed diagnoses of major infection and admission diagnoses of major infection; pulmonary embolism (PE; 0.19%), which included confirmed diagnoses of PE and admission diagnoses of PE; deep vein thrombosis (DVT; 0.18%), which comprised confirmed diagnoses of DVT and admission diagnoses of DVT; and finally hypoxia (0.11%).

### Combination Procedures

Of the patients who underwent an abdominoplasty, 15,124 also had concurrent surgeries related to the breast, body contouring, liposuction, or the face. The majority of these patients had breast-related surgeries (*n* = 10,549, 69.7%). The second most common combination procedure was body contouring, with nearly 3000 patients (19.2%) receiving this combination of procedures. There was a significant difference in the overall rate of complications between combination procedures and abdominoplasty alone (*P* = .021). However, when accounting for various factors in our regression model, there was no significant added risk for major complications after undergoing a combination procedure compared to an abdominoplasty alone (Table 5). Overall complication rate for combination procedures are reported in Table 6.

**Table 6.** Complication Rates of Combination Procedures

Abdominoplasty plus:	Number of complications	Complication rate (%)
Breast (*n* = 10,549)	244	2.31
Body contouring (*n* = 2902)	74	2.55
Liposuction (*n* = 923)	18	1.95
Face (*n* = 516)	14	2.71
Other (*n* = 234)	4	1.71

## DISCUSSION

Abdominoplasty has become one of the most popular cosmetic surgical procedures in the past decade. Techniques and variations of the conventional procedure have developed, and patients and surgeons now have many options to pursue. In this study we investigated the complication rates and risk factors associated with the different types of abdominoplasty procedures. There was a significant difference among the overall complication rates for all 7 procedures, with fleur-de-lis abdominoplasty having the highest overall complication rate (3.81%). Previous works have also shown significant differences in major and minor complications related specifically to fleur-de-lis abdominoplasty, and our work demonstrates similar results with regard to major complications, with an overall major complication rate of 3.81% in the current study, compared to that of previous work showing a major complication rate of 5%.^19^ The reported complication rate in the literature for abdominoplasty is extremely varied and can range from 3% to 51.8%, depending on the type of abdominoplasty being measured.^14-18,20,21^ However, many of these studies included a very small sample size and cannot be accurately translated to the broader population. The present study provides a comprehensive review of postoperative outcomes of different types of abdominoplasties performed by hundreds of private practice and academic board–certified or board-eligible plastic surgeons from across the US, substantially adding to the previous literature.

When investigating risk factors for each type of abdominoplasty, an interesting and paradoxical finding of our analysis was that patients who are underweight (BMI < 18) or with morbid obesity (BMI > 40) had a significantly higher risk of developing a complication with a conventional abdominoplasty, compared to patients who have a normal BMI (OR, 21.50 and 1.70, respectively, *P* < .05). Current literature outlines a significantly increased risk for complications in overweight patients, those with obesity or morbid obesity, and in patients who are underweight.^4,14,17,22,23^ Other specialties have literature that exhibits the same bimodal finding, with extremes of BMI demonstrating higher complication rates, such as the orthopedic joint replacement literature, as well as the spine surgery literature.^24-26^ The general surgery literature demonstrates higher rates of mortality among underweight patients undergoing cholecystectomy, and the vascular surgery literature supports higher rates of mortality in patients with peripheral artery disease, with the mechanisms for this finding still being elucidated.^27,28^ Our results demonstrate that obesity remains a risk factor for major complications in the morbidly obese as well as in individuals who are underweight, suggesting that this demographic seek additional preoperative risk modification, if possible, before undergoing abdominoplasty surgery. Further, the interesting and paradoxical nature of underweight patients having higher rates of complications after abdominoplasty, as has been seen in multiple other surgical disciplines, raises this as an important area for future study.

Considering the increase in popularity in what in the mainstream vernacular is known as the “mommy makeover,” it is important to note the complication rates in combination procedures. Generally, this common combination procedure includes an abdominoplasty with a breast surgery (typically augmentation, mastopexy, augmentation-mastopexy, or reduction), with or without liposuction. Our analysis demonstrated that there was no significant added risk for developing major complications between having an abdominoplasty and a breast, body contouring, liposuction, or facial procedure when compared to having an abdominoplasty alone. Furthermore, an abdominoplasty and a breast procedure, as in the “mommy makeover,” was the most common procedure combination performed. The complication rate for these procedures is similar to some previous reports in the literature that showed complication rates for the combination procedure being similar to the individual staged procedures (combined: 34% vs abdominoplasty alone: 10%-40% and cosmetic breast alone: 2%-25%).^29^ This observation stands in contrast to our previously published work, which showed significant increased risk with the addition of other procedures to an abdominoplasty—a risk increase that was significantly additive with each additional procedure. An important difference between our previous study and the current cohort is the disparate numbers of the complements of abdominoplasty performed solo vs combined procedure. Interestingly, in our previous work, of the 25,478 patients who underwent abdominoplasty, 64.8% (16,503) had abdominoplasty combined with another procedure, whereas the current data set of more than double the overall patients undergoing abdominoplasty (55,596) had only 27.2% (15,124) undergoing concomitant procedures. In comparing the 2 cohort data sets, it appears that plastic surgeons have amended their practice patterns to perform fewer procedures combined with abdominoplasty to offer safer cosmetic procedures to their patients, as evidenced by the now nonsignificant risk for increased complications in abdominoplasty combined with additional procedures.^15^

The year surgery was performed also presented interesting results. In terms of procedure count, our results demonstrated a spike in cases from 2020 to 2021. In 2020, nearly 7000 patients underwent an abdominoplasty, whereas in 2021 this increased to 10,598 patients. At the onset of the COVID-19 pandemic, there was a period of time at which elective procedures had to be postponed to help relieve the operational pressure placed on hospitals by the historical influx of patients needing medical assistance.^30^ A survey administered to plastic surgeons at the height of the pandemic revealed that less than 10% of respondents were offering aesthetic procedures.^29^ Additionally, another survey administered to plastic surgeons by the American Society of Plastic Surgeons reported that following the pandemic patients were more motivated to seek cosmetic surgeries for various reasons, including paying for the surgeries with travel budgets, wanting to feel good following the pandemic, and remote work making recovery easier.^2^ The increase in the number of abdominoplasties in 2021 and 2022 could be attributed to this. We found it interesting to see that complication rates for nearly every type of abdominoplasty were significantly higher in the early part of the data set—during the 3 years from 2017 to 2019, and then were significantly lower the following 3 years, from 2020 to 2022. Although it is difficult to demonstrate a causative effect for the year of surgery and the associated risk for complications in abdominoplasty, it is interesting to see the correlated nonsignificance of complications in abdominoplasty patients for the final 3 years of this cohort study period, compared to significant values for each type of abdominoplasty performed in the previous years. It is possible that fewer combined procedures with abdominoplasty plays a pivotal role in this effect when making the comparison. As described in our discussion of mommy makeovers and abdominoplasty with combined procedures, more data clarifying safe surgery in abdominoplasty and combined procedures has emerged over time, and fewer combined procedures were seen in this cohort (27.2%) compared to our study in 2015 (64.8%). We intend to further delve into this interesting finding of improved outcomes over time in our group’s future study, because it importantly suggests that board-certified plastic surgeons are continuing to improve outcomes and offer safer surgical procedures for their cosmetic surgery patients.

There are many strengths to this study. To our knowledge, this is the first study reporting on evaluation of complication rates and associated risks of different types of abdominoplasties performed employing a large, prospectively maintained, national database. In addition, we report information on granular demographic factors including year of surgery, facility type, type of anesthesia, and the ASA physical status classification system. This detailed analysis provides greater information on additional factors that may affect a patient's outcome than has yet to be formally reported in the literature. The prospective nature of the CosmetAssure database provides a unique ability to obtain baseline information on the number of patients receiving the procedures investigated, allowing for a more robust analysis and conclusion. This robust data set has been validated in multiple previous studies.

As with any study, there are limitations worth noting. Because CosmetAssure is an insurance company, the complications recorded in their database only include major surgical complications. Information about minor complications is not available in the data set. Minor complications are significantly more common than major complications and are important in cosmetic outcomes and the results that patients experience. In addition, the patient population in this study is limited to patients of plastic surgeons who participate in the CosmetAssure program. The data do not include patients who undergo an abdominoplasty by surgeons whose patients cover treatment for any postoperative complication with other methods. These data show the outcomes for cosmetic abdominoplasty and associated low complication rates, as well as the risk factors for those complications when the procedure is performed by board-certified or board-eligible plastic surgeons at accredited facilities. This high standard for cosmetic care likely imparts a selection bias when comparison is made to patients undergoing abdominoplasties by other surgeons and at other facilities without such qualifications.

## CONCLUSIONS

Abdominoplasty is one of the most common cosmetic procedures performed in the United States. We demonstrate in this study that overall complications for abdominoplasty are low at 2.1%, and the risk for complications in abdominoplasty has decreased over time when compared to previous years. However, there exists a significant difference in the overall complication rates between the different types of abdominoplasties, with fleur-de-lis abdominoplasty having the highest overall complication rate among the 7 types of abdominoplasties studied (3.81%). There was no difference in complication rates when an abdominoplasty was combined with other types of cosmetic surgical procedures. Body mass index, diabetes, and gender place patients at a higher risk for developing a major postoperative complication.
